# The clinical and cost-effectiveness of a Victim Improvement Package (VIP) for the reduction of chronic symptoms of depression or anxiety in older victims of common crime (the VIP trial): study protocol for a randomised controlled trial

**DOI:** 10.1186/s13063-020-4211-9

**Published:** 2020-04-16

**Authors:** Marc Serfaty, Trefor Aspden, Jessica Satchell, Anthony Kessel, Gloria Laycock, Chris R. Brewin, Marta Buszewicz, Aidan O’Keeffe, Rachael Hunter, Gerard Leavey, Jon Cuming-Higgs, Vari Drennan, Monica Riveros, David Andrew, Martin Blanchard

**Affiliations:** 1 Division of Psychiatry, UCL, 6th Floor Maple House, 149 Tottenham Court Road, London, W1T 7NF UK; 2 Public Health England, 133-155 Waterloo Road, London, SE1 8UG UK; 3 Jill Dando Institute of Security and Crime Science, UCL, 35 Tavistock Square, London, WC1H 9EZ UK; 4 Clinical Psychology, UCL, Gower Street, London, WC1E 6BT UK; 5 Research Department of Primary Care and Population Health, UCL, Gower Street, London, WC1E 6BT UK; 6 Department of Statistical Science, UCL, Gower St., London, WC1E 6BT UK; 7 Research Department of Primary Care and Population Health, UCL, Royal Free Medical School, London, NW3 2PF UK; 8 Bamford Centre for Mental Health Wellbeing, Ulster University, Cromore Road, Coleraine, Northern Ireland; 9 Mind, 15-19 Broadway, London, E15 4BQ UK; 10 Joint Faculty of Health, Social Care and Education, Kingston University and St. George’s University of London, Cranmer Terrace, London, SW17 0RE UK; 11 Age UK Camden, Tavis House, 1-6 Tavistock Square, London, WC1H 9NA UK; 12 Lived experience researcher/user representative Middlesex, London, UK; 13 UCL, Gower St., London, WC1E 6BT UK; 14 Priory Hospital North London, London, N14 6RA UK

**Keywords:** Older victims, Crime, Anxiety, Depression, CBT

## Abstract

**Background:**

Older people are vulnerable to sustained high levels of psychosocial distress following a crime. A cognitive behavioural therapy (CBT)-informed psychological therapy, the Victim Improvement Package (VIP) may aid recovery. The VIP trial aims to test the clinical and cost-effectiveness of the VIP for alleviating depressive and anxiety symptoms in older victims of crime.

**Methods/design:**

People aged 65 years or more who report being a victim of crime will be screened by Metropolitan Police Service Safer Neighbourhood Teams within a month of the crime for distress using the Patient Health Questionnaire-2 and the Generalised Anxiety Disorder-2. Those who screen positive will be signposted to their GP for assistance, and re-screened at 3 months. Participants who screen positive for depression and/or anxiety at re-screening are randomised to a CBT informed VIP added to treatment as usual (TAU) compared to TAU alone. The intervention consists of 10 individual 1-h sessions, delivered weekly by therapists from the mental health charity Mind.

The primary outcome measure is the Beck Depression Inventory-II (BDI-II) and the Beck Anxiety Inventory (BAI), used as a composite measure, assessed at 6 months after the crime (post therapy) with a 9-month post-crime follow-up. Secondary outcome measures include the EQ-5D, and a modified Client Service Receipt Inventory. A total of 226 participants will be randomised VIP:TAU with a ratio 1:1, in order to detect a standardised difference of at least 0.5 between groups, using a mixed-effects linear-regression model with 90% power and a 5% significance level (adjusting for therapist clustering and potential drop-out).

A cost-effectiveness analysis will incorporate intervention costs to compare overall health care costs and quality of life years between treatment arms. An embedded study will examine the impact of past trauma and engagement in safety behaviours and distress on the main outcomes.

**Discussion:**

This trial should provide data on the clinical and cost-effectiveness of a CBT-informed psychological therapy for older victims of crime with anxiety and/or depressive symptoms and should demonstrate a model of integrated cross-agency working. Our findings should provide evidence for policy-makers, commissioners and clinicians responding to the needs of older victims of crime.

**Trial registration:**

International Standard Randomised Controlled Trials Number, ID: ISRCTN16929670. Registered on 3 August 2016.

## Background

Anyone can be a victim of crime. Its behavioural and psychological effects on quality of life can be severe [1]. Older people may be particularly vulnerable to the effects of crime because of concurrent life events, such as family bereavements, diminishing support networks, increasing physical frailty or financial difficulties [2–5]. Consequently, older people may feel more isolated, more vulnerable, and require additional support following a crime [5].

Our society continues to age, with people aged 85 years or over estimated to reach 3.2 million in the UK by 2033 [6]. From the limited data on the impact of crime in older people, there appear to be increases in psychological distress, social care needs and mortality. Older victims of crime have significant levels of depression and anxiety [4, 7, 8] and are at increased risk of needing placement in a care home [9], or death [10], compared to their peers who have not experienced a crime. Psychological morbidity also compounds age-associated ill-health and disability, leading to higher use of social and health care services [11].

Currently, 95% of depressed older people receive no specific treatment, with only 5% being referred to mental health services compared to 50% of younger adults, and only 3.7% of referrals for psychological therapies being for older people [12]. Failure to treat depression and anxiety in older people often leads to chronicity of their symptoms for months or years [13].

There is scant research regarding specific interventions for older people who have been victims of crime. One small study of a video-based intervention for those with anxiety or depressive symptoms showed no significant benefit [14]. Our National Institute for Health Research-funded study ‘Helping Aged Victims of Crime’ (HAVoC) [8], which assessed the impact of crime and its management in older people, confirms its significance as a public health issue: 3 months after a crime, 65% of victims felt that it had affected their daily life and 27% were psychologically distressed, with just under a half meeting *Diagnostic and Statistical Manual of Mental Disorders 4th Edition* (*DSM-IV*) criteria for a psychiatric disorder attributed to the crime. Findings from the HAVoC study [8] suggest that our specially tailored cognitive behavioural therapy (CBT)-informed psychological therapy, the Victim Improvement Package (VIP) may benefit people with depressive- and anxiety-related symptoms. Findings from the HaVoC study informed the present, fully powered randomised controlled trial (RCT) to test the clinical and cost-effectiveness of the addition of a psychological therapy (the VIP) to usual care, compared to usual care alone.

### Objectives

The objectives of this study are as follows:

1. *Primary objective*: to determine whether older victims with depression and/or anxiety present 3 months after a crime benefit from a VIP to reduce continued severity of symptoms 6 months post crime

Secondary objectives were:

2. *Symptom severity*: as above, but measured at 9 months post crime (follow-up)
3. *Quality of life*: to determine whether older victims benefit from a VIP in terms of improving quality of life at 6 and 9 months post crime
4. *Economic*: to determine the cost-effectiveness of the VIP
5. *Health inequalities*: to explore the impact of the VIP on health inequalities
6. *Signposting*: to explore the effectiveness of signposting to primary care
7. *Service delivery*: to demonstrate an effective model for identifying, referring and treating older victims of crime suffering from depression and/or anxiety

## Methods/design

### Study design

This trial will use mixed quantitative and qualitative methods as recommended by the Medical Research Council’s guidelines on for complex interventions [15]. The main trial is a parallel-group, single-blind, individually randomised controlled trial comparing treatment as usual (TAU) against TAU plus up to 10 sessions of a VIP. Randomisation will be stratified by anxiety alone or depression with or without anxiety.

### Ethical approval

This trial has been approved by the University College London (UCL) Ethics Committee (project No. 6960/001), and is sponsored by UCL. The trial will be conducted in compliance to the Declaration of Helsinki. Informed written consent will be obtained from all participants.

### Population

The study population consists of victims aged 65 years or more with symptoms of anxiety and/or depression attributed to being the victim of a crime (specific crimes included are outlined in the entry criteria below).

### Location

Participants will be recruited from nine London boroughs: Hackney, Tower Hamlets, Camden, Islington, Barnet, Havering, Haringey, Newham and Enfield. These areas were selected for demographic diversity (ethnicity, social economic status) and because of the availability of local psychological services, provided by the mental health charity Mind.

### Recruitment methods and procedures

Recruitment into this RCT follows a three-step procedure, comprising: (1) screening; (2) re-screening; and (3) the RCT. These three steps are outlined below:

- Step 1

*Screening.* Any person aged 65 years or more, recorded as a victim of crime (excluding domestic and sexual crimes) from one of nine London boroughs will be identified from a computer search on the Metropolitan Police Service (MPS) database. Identified victims will be contacted by a police administrator to arrange a visit (either a police community support officer or a police constable), from one of the MPS Safer Neighbourhood Teams (SNTs) within a month of the crime.

A pre-populated proforma completed by a central administrator will be forwarded to a police officer who then visits to explain the trial to victims. Informed consent to participate will be sought by the visiting officer. If the person agrees, the officer will collect additional demographic information and details about the crime as well as screening the victim for depressive and anxiety symptoms using abbreviated forms of the Patient Health Questionnaire (PHQ-2 [16]) and Generalised Anxiety Disorder Scale (GAD-2 [17]), respectively (see Table 1). Victims who score 3 or more on the PHQ-2 and/or 2 or more on the GAD-2 are considered screen-positive; scores below these cut-offs on both of these measures are case-negative. The police will then signpost the screen-positive participants to their general practitioner (GP), providing an accompanying letter that states that the person appears to be experiencing significant distress, recommending that the GP should manage them as they see fit. Those who are screen-negative are provided with information about the impact of crime and advised to see their GP if they develop symptoms of anxiety or depression.

**Table 1:** Standard Protocol Items: Recommendations for Interventional Trials (SPIRIT) diagram showing trial time points

Summary of main measures (all with reference to post crime)	Post-crime eligibility check	Enrolment	Post allocation			Closeout
	0 (post crime)	3 months	Baseline 3 months	Post intervention 6 months	Follow-up 9 months	
**Enrolment:**						
Post-crime eligibility screen	X					
Baseline eligibility screen		X				
Informed consent		X				
Allocation		X				
**Interventions:**						
Victim Improvement Package			X			
Treatment as usual			X			
**Assessments:**						
BDI-II			X	X	X	
BAI			X	X	X	
MINI (caseness)/specific anxiety disorder			X	X		
EQ5-D			X	X	X	
CSRI			X	X	X	
Childhood Trauma Questionnaire			X			
Safety Behaviour Questionnaire			X	X	X	
Satisfaction with VIP				X		
Expectation of therapy			X			
Blindness assessment by research assistant				X	X	
Attrition and reason				X	X	
Fidelity: adherence and CTS-R				X		

*BAI* Beck Anxiety Inventory, *BDI-II* Beck Depression Inventory-II, *CSRI* Client Service Receipt Inventory, *CTS-R* Cognitive Therapy Scale-Revised, *EQ-5D* EuroQol 5 dimensions health survey, *MINI* Mini-International Neuropsychiatric Interview, *VIP* Victim Improvement Package

The trial will start with SNTs screening all suitable older victims for distress. However, as police resources are constrained, once we have established that SNTs can screen both positive and negative older victims, procedures will be adapted to screen older victims over the phone and only for SNTs to visit those who are screen-positive or deemed vulnerable. This will ensure that police resources are targeted at distressed older victims only.

In addition to the above a subset of screen-positive and screen-negative victims from steps 1 and 2, are invited to take part in qualitative interviews. Details of these are outlined in the ‘Qualitative research’ section below.

- Step 2

*Re-screening*. Screen-positive victims from step 1 are re-screened 3 months after the crime over the phone for continued symptoms of depression (≥ 3 on PHQ-2) and/or anxiety (≥ 2 on GAD-2). Eligibility criteria (outlined below) are assessed. A home visit by a trial researcher further assesses participants to ensure that they satisfy all the entry criteria for the RCT (step 3).

- Step 3

*RCT*. Those satisfying entry criteria are given a copy of the participant information sheet for the RCT, and given at least 48 h to consider and discuss its contents prior to any request for informed consent to participate in the trial.

### Eligibility criteria

Inclusion criteria are:

1. Victim of a reported crime including common assault, actual bodily harm, grievous bodily harm, harassment, racist crime, homophobic crime, false representation (deception), burglary, distraction burglary, criminal damage to property, theft including pick-pocketing and snatch.
2. Age 65 years or older
3. Symptoms of depression (indicated by a score of 3 or more on the PHQ-2 [16]) and/or symptoms of anxiety (indicated by a score of 2 or more on the GAD-2 [17])

Exclusion criteria are:

1. Having ever been diagnosed with schizophrenia or bipolar disorder (assessed with yes/no questions). These diagnoses are not targeted by the VIP intervention and could affect outcome
2. A Mini-International Neuropsychiatric Interview (MINI [18]) diagnosis of alcohol dependency
3. Receipt of CBT in the last 6 months
4. Inability to participate in CBT because of insufficient proficiency in the English language
5. Significant cognitive impairment, indicated by a six-item Cognitive Impairment Test [19] score of 10 or more
6. High suicide risk determined by the MINI

### Risk management

For people deemed to be a high suicide risk on the MINI, our protocol ensures safety by notifying the participant’s GP, a relative or, in rare cases, the police so that a further assessment may be sought about risk. The researcher will remain with the participant until additional help is available. A discussion with the chief investigator also takes place to determine whether they are suitable for the trial.

### Randomisation

Participants who consent to enter the RCT and complete baseline measures (step 3 of the study, 3 months post crime) are randomised to one of two conditions: TAU or TAU plus the VIP. Randomisation is conducted by the trial administrator using a web-based randomisation service, Sealed Envelope, provided by PRIMENT Clinical Trials Unit, an independent United Kingdom Clinical Research Collaboration registered clinical trials unit. This randomisation system has been pre-populated with a randomised list provided by an independent PRIMENT statistician and uses random permuted blocks of variable size to stratify participants by their primary diagnosis (anxiety alone or depression with or without anxiety).

Attempts will be made to ensure that all researchers collecting outcome measures are kept blind to the group allocation.

### Descriptions of the interventions

#### The Victim Improvement Package (VIP)

##### Therapist background and training

Therapists from a major national mental health charity (Mind), previously trained in CBT techniques and with at least 2 years’ experience in delivering CBT will be the main target for therapist selection, being chosen to be at a standard accreditable by the British Association of Behavioural and Cognitive Psychotherapies (BABCP). However, we will be constrained by the availability of therapists and will adapt a pragmatic approach, using therapists who are experienced in psychological approaches and have undertaken training in CBT. All therapists will be given a full day of training on how to apply their existing skill set to the VIP intervention. They will be taken through the VIP manual step by step and also asked to participate in role plays of various scenarios typical of older victims of crime.

##### Delivery of therapy

Up to 10 manualised [20] individual sessions of CBT especially modified for older crime victims will be delivered over 3 months, in community-based Mind facilities. If the victim has a preference to be treated in their own home; for example, due to physical disability or if they fear leaving the house after the crime, they can, at the discretion of the Mind therapist, receive the therapy in their home. In this instance the local Mind centre’s lone-working policy will be adhered to at all times while providing home therapy.

##### Content of therapy

Full details of the VIP manual have been published and are available from the chief investigator [20]. The VIP, tailored to the main presenting symptoms and used flexibly, will cover: Session 1: a narrative of the crime, underlying beliefs, behaviours and how these have changed; Session 2: psycho-education about crime and an introduction to CBT; Sessions 3–8: mood diaries to identify unhelpful thinking and behaviours; guided discovery to challenge beliefs about crime, personal vulnerability and safety; behavioural experiments to challenge unhealthy avoidance; Sessions 9–10: relapse prevention. Therapy sessions will follow a typical CBT model: setting an agenda, conducting therapy and encouraging between-session homework exercises.

#### Treatment as usual (TAU)

Historically, all older crime victims were offered a visit from a SNT, which is usual care for the purpose of the trial. However, changes in resources suggest that policing will shift to SNTs undertaking selected ‘cocooned’ visits to victims (burglary and those deemed vulnerable by the MPS). Informal support may be provided by networks of friends and relatives, where available.

##### Voluntary agencies

All victims are routinely provided with information on how to contact Victim Support. This relies on the victim proactively requesting assistance, which older people do not usually appear to do [8]. People may also self-refer to Mind. In reality, few older people take up offers of help when contacted by letter [8].

##### GP referral

Concerning older people, our previous qualitative work found that victims do not usually seek help from their GP directly, as they do not believe that the problem is ‘medical’. When referred to the GP with depressive and anxiety symptoms they may not be managed according to best practice as indicated in the National Institute of Health and Care Excellence (NICE) guidelines, especially when distress is seen as understandable or associated with ageing [12]. A small number may be prescribed psychotropic medication, but many are reluctant to take these [21]. With anti-depressant use there may be difficulties with compliance, fears of dependence, interactions and side effects.

##### Psychotherapy referral

A recent development in England has been the Improving Access to Psychological Therapies (IAPT) service where people may self-refer, or be referred by their GP. These IAPT services use a stepped-care model with provision for limited CBT. CBT is recommended by NICE as an effective treatment for anxiety and depression in people of all ages, but generally very few older people receive this [12]. We will not exclude the possibility of participants receiving CBT in the TAU arm for ethical reasons, but we will record any receipt of this and account for it in the analysis.

Traumatic Stress Clinics: these rarely see older adult victims and, as they are tertiary services, it may take people at least a year to gain access to treatment.

Access to independent practitioners: for economic reasons, older people are less likely to pursue privately financed options.

Where the participant requests it, their GP will be informed about the study and their patient’s symptoms. We will not encourage referral for CBT or starting or increasing psychotropic medication during the trial.

### Outcomes

#### Primary outcomes

The Beck Depression Inventory-II (BDI-II) [22] and Beck Anxiety Inventory (BAI) [23] are used for those diagnosed with depression and anxiety, respectively, to measure participants’ progress. These two scales will be adjusted to enable us to compare improvements in depression or anxiety using ‘standardisation’. For each outcome (BDI-II and BAI), standardisation entails subtracting the corresponding sample mean and dividing by the corresponding sample standard deviation, to produce a composite outcome for all participants (whether anxious or depressed). Both the BDI-II and BAI are self-reported scales with 21 items each having four possible answer choices. They have good reliability and validity for measuring severity of depression and anxiety, respectively. The BDI-II has the advantage over other scales because it includes a significant number of cognitive-affective as well as somatic dimensions. The BAI is composed of cognitive and somatic elements.

#### Secondary outcomes

##### Diagnostic category

The Mini-International Neuropsychiatric Interview (MINI) [18] is used to assist diagnostically, as it generates a *Diagnostic and Statistical Manual of Mental Disorders, 4th edition* (*DSM-IV*) diagnosis of at least one of the following (we have included the *equivalent International Classification of Diseases 10th edition* (*ICD-10)* codes in brackets): depression with or without anxiety, DSM 296.2x, 296.3x, (*ICD-10* F32.x); panic disorder without agoraphobia 300.01 (*ICD-10* F41.0) or with agoraphobia 300.21 (*ICD-10* F41.0); generalised anxiety disorder *DSM-IV* 300.02 (*ICD-10* F41.1); agoraphobia without history of panic disorder *DSM-IV* 300.22, (*ICD-10* F41.1). Although the MINI does not generate a diagnosis of specific anxiety disorder *DSM-IV* 300.09 (*ICD-10* F41.8), which is relevant to our target population, this diagnostic group will also be included in the study if participants experience significant anxiety attributable to the crime.

The *EuroQol 5 dimensions health survey (EQ5-D; Rabin 2001)* is a five-item generic utility measure of quality of life. It has been selected because: (1) It is brief, easy to use, and minimises attrition; (2) It compares favourably with other measures [24, 25]; (3) It has been used extensively with older people [26]; (4) We have used it with depressed older people [27, 28] and older victims of crime [8]; and (5) It is recommended by NICE for health economics [29], including trials with older people [30].

The *Client Service Receipt Inventory: (CSRI; Curtis 2008)* a modified version of the CSRI, developed for the present study, will be used to collect data on service use to inform the health economic analysis.

*Generalised Anxiety Disorder Assessment (GAD-7) and Patient Health Questionnaire (PHQ-9)*. The PHQ-9 and the GAD-7 are valid measures of depression and anxiety, respectively [31, 32], which are widely used in primary care settings.

### Other measures

#### Childhood Trauma Questionnaire

The Childhood Trauma Questionnaire Short Form (CTQ-SF [33];) is a 28-item self-report screening tool that can be used in both adults and adolescents to detect quickly five types of childhood maltreatment: physical, emotional and sexual abuse, and physical and emotional neglect. The CTQ-SF has been selected because it is the most commonly used measure to assess multiple maltreatment types [34] and studies support its reliability, validity and factor structure [34, 35]. The measure has also been used in studies with older adults (e.g. [36]).

#### A measure of safety behaviours

Safety behaviours (also called safety-seeking behaviours) are counterproductive and dysfunctional overt or covert actions intended to prevent, escape from, or reduce the severity or risk of a potentially threatening outcome [37, 38]. A measure is being developed as part of the VIP trial to collect data on safety behaviours in older crime victims. Participants are prompted to provide their own examples of excessive checking, reassurance-seeking, rumination, avoidance, compulsions, hypervigilance or any other behavioural changes. They are then asked to rate on a 7-point Likert scale how frequently they engage in these behaviours and how much this has changed since the crime happened.

#### Measures of sources of bias during the course of the trial

##### At baseline (3 months post crime)

1. *Prescribed anti-depressants and/or anxiolytics and/or hypnotics*: participants are asked if they are taking any medication to help with mood, depression, anxiety or sleep. They are also asked the name, dose and length of prescription. Doses will be standardised against fluoxetine/diazepam, respectively, to see whether they are equivalent in both trial arms
2. *Other psychological therapies* reported by participants
3. *Expectations at baseline*: participants will be asked to predict the degree of expected improvement, or not, on a Likert scale ranging from 0 to 10
4. *Treatment preference*: participants’ treatment group preference (VIP group/TAU group/no preference) will be recorded at baseline
5. *Cancelled/missed therapy sessions*

##### Post therapy (6 months)

1. *Measures of attrition and engagement with therapy*: during the course of the study we expect a few participant deaths, which are likely to be random. Additionally, we will record change of residence, illness, geographical distance from therapy, did not attend rates, and the reasons for not doing so
2. *Assessment of ‘blindness’ by rater*. At the primary endpoint of 6 months, once the BDI-II and BAI have been rated, the researcher will be asked to guess the group allocation (TAU or VIP or not sure)
3. *Changes in prescribed psychotropic medication:* anti-depressant or anxiolytic/hypnotic medication
4. *Other psychological treatments received*. This may include seeing a counsellor, psychotherapist or victim support counsellor and can include both statutory and privately funded provision
5. *Measures of satisfaction with treatment* by rating on a 5-point scale (*not at all* to *very much*) whether the VIP was perceived as useful
6. *Reason for missing follow-up (e.g. withdrew, died, etc..*

#### Timing of enrolment, interventions and measures

The timing of enrolment, allocation, collection of measures and study closeout, is summarised in the Standard Protocol Items: Recommendations for Interventional Trials (SPIRIT) diagram in Table 1.

#### Assessment of therapists’ competence and adherence to VIP manual

We encourage all therapists to digitally record their therapy sessions and a random sample of 1 in 10 recordings will be selected for quality control for competence of delivery of the therapy and adherence to the manual.

##### Therapists’ competence

Treatment quality will be rated by an accredited member of the British Association of Behavioural and Cognitive Psychotherapies expert in the field using the Cognitive Therapy Scale-Revised [39]. Each item is rated from 0 to 6 on a visual analogue scale, ranging from incompetent through to novice, advanced beginner, competent, proficient and expert. The total score ranges between 0 and 72. Therapists would be expected to achieve a minimum score of 36.

##### Adherence to the VIP manual

The use and the development of a Therapy Components Checklist (TCC) arose from previous work [27, 40] which was further developed in the HAVoC study [8]. We identified what we considered to be 26 core elements required in the delivery of the VIP. These core elements were operationalised in the treatment manual. Central to the components checklist, we wanted to ensure that the therapists adhered to the range of CBT procedures (i.e. they used a combination of cognitive, behavioural and cognitive-behavioural techniques, as well as specific issues related to our CBT intervention for victims of crime). The therapists will be asked to list the components of therapy that they have covered at the end of each therapy session. We will describe the components reported, requesting the independent assessor responsible for rating therapists’ competence to complete the TCC for the rated session and compare the therapists’ self-report with their assessors report.

##### Therapist supervision

All therapists will be asked to phone at least once a month to the group supervision taking place twice monthly. They will be asked to present their therapeutic interventions that worked well as well as identifying problem areas. They will also be able to contact the chief investigator by phone, or by email with a response within two working days, to discuss any major therapeutic difficulties.

#### Data entry and storage

Outcome data will be collected directly onto paper forms by researchers employed on the VIP trial. Each paper form will then be hand-delivered to the main trial office by the trial researcher. This data will then be entered onto the secure online Sealed Envelope database by a member of the trial team (either a trial researcher or trial administrator). A portion (10%) of all entered data will be checked against the original paper form for accuracy. At the end of the trial after database lock, the trial dataset will be downloaded from Sealed Envelope by the trial statisticians.

#### Trial monitoring

The trials Data Monitoring Committee comprises three independent members including an expert in clinical trials methodology and medical statistics, an expert in mental health, and an expert in old-age psychiatry. This Data Monitoring Committee will meet at least once a year throughout the running of the trial. A Trial Steering Committee will also meet once a year. The Trial Management Group, consisting of grant applicants, public representatives, and members of the clinical trials unit PRIMENT, will meet approximately every 3 months throughout the running of the trial. Both the Trial Steering Committee and the Data Monitoring Committee contain members who are independent of the trial, and will be able to hold closed sessions without trial members present to independently monitor the trial and make any necessary decisions on the running of the trial.

As no harm to participants is regarded as likely from CBT, the trial does not have specific interim analysis or stopping guidelines. The Data Monitoring Committee and Trial Steering Committee will have the usual power to stop the trial if they deem this necessary, as outlined in the National Institute for Health Research guidelines for trial committees.

#### Statistics

##### Power

Efficacy analysis will use changes from baseline to the end of the intervention period (6 months post crime) in a standardised combination of BAI and BDI-II scores. For standardisation, the corresponding sample mean (BAI or BDI-II) will be subtracted from the BAI or BDI-II measurement and then the resulting measure divided by the corresponding sample standard deviation (BAI or BDI-II). This will yield a standardised outcome measure to be modelled for all trial participants (whether anxious or depressed, with or without anxiety). Although there is little evidence as to what constitutes a clinically meaningful difference [41], consensus between experts in the field suggest that a change of 0.5 of a standard deviation, 3 or more on the BDI, is considered a NICE-approved clinically important change [42]. It is feasible to detect a (‘true’) average difference of at least 0.5 on the standardised joint scale with 90% power and a 5% significance level using a total sample size (*N*) of 168, with participants randomised VIP:TAU with a ratio 1:1. Applying an overall ‘cluster-adjustment’ for therapist effects, assuming a cluster-size of 8 intra-class correlation coefficient (ICC) of = 0.02 and 15% allowance for drop-out, increases this to *N* = 226. Using data from the pilot study, the ‘target’ standardised difference of 0.5 implies changes in both BAI and BDI-II of about 4 and this is valuable clinically, given the scales show that for moderate levels of symptoms the scores range from 20 to 28 and 16–25 for anxiety and depressive symptoms, respectively.

##### Clinical-effectiveness analysis

We will follow a pre-specified plan for statistical analysis and reporting which will be finalised before database lock, and in adherence with Consolidated Standards of Reporting Trials (CONSORT) guidelines.

The primary analysis will be based on available data and conducted according to the intention-to-treat principle. The mean difference in the primary outcome in the VIP group compared to the TAU group at this time point will be estimated from a mixed-effects linear-regression model with standardised BAI/BDI-II score at 6 months post crime as the response variable, and with study arm, centre and baseline standardised BAI/BDI-II score as explanatory variables. The model will account for clustering by Mind therapist in the VIP arm using a therapist-level random effect and a patient-level random effect to account for repeated measures within patients.

Participants will be classified as having either ‘anxiety’ (alone) or ‘depression’ (with or without anxiety) and randomisation will be stratified by this primary diagnosis using separate analyses, comparing TAU plus VIP vs TAU.

The primary outcome for participants in the depression stratum is BDI-II score whereas the primary outcome for participants in the anxiety only stratum is BAI score. A combined primary outcome for both strata will be created in the following manner:

- BDI-II scores will be standardised by the mean and standard deviation of BDI- II score at baseline of all participants in the depression stratum irrespective of allocation
- BAI scores will be standardised by the mean and standard deviation of BAI score at baseline of all participants in the anxiety-only stratum irrespective of allocation
- The standardised versions of BDI-II scores from the depression stratum and BAI scores from the anxiety-only stratum will then be combined into a single variable to form the primary outcome measure, used in the linear mixed-effects regression model

##### Supportive analyses

Bias due to missing data will be investigated by comparing the baseline characteristics of participants with and without missing values. Depending on the quantity of missing values, the predictors of missingness will be identified. We will then perform a sensitivity analysis by including any predictors of missingness as explanatory variables in the primary outcome model.

If appropriate, we will also perform a multiple imputation analysis [43] in which we will construct a model to impute missing BDI-II/BAI post intervention (6 months post crime) using appropriate explanatory variables. To account for potential differential attrition between socio-economic groups, Lower Layer Super Output Area (LSOA) data (an indicator of relative deprivation) will be part of the predictor in the multiple imputation model. We will also consider baseline BDI-II/BAI score, participant demographics recorded at baseline, and any other variables thought to be related to missingness.

*Exploratory analyses:* will be carried out to describe how a limited number of pre-specified characteristics of participants may modify treatment effects. These will include patient preferences, relative levels of deprivation (LSOA data) and non-compliance with treatment: the latter being addressed using compliers’ average causal effects (CACE) analysis.

##### Secondary outcome variables

Analyses of secondary outcomes will be performed using models analogous to those described in relation to the primary outcome. Continuous outcomes will be analysed using mixed-effects linear-regression models. Binary outcomes will be analysed using mixed-effects logistic regression models. Model parameter estimates will be reported accompanied by 95% confidence intervals.

### Qualitative data

Semi-structured interviews will be completed with around 25 older crime victims at step 2 of the trial. The topic guide will explore three key areas: (1) the social and cultural background of the victim and how this influences their perception of the crime; (2) safety attitudes and behaviours following the crime event; and (3) their experience of services and help-seeking since the crime. A purposive sample will be sought in order to achieve a balance of socio-demographic characteristics. The interviews will be analysed using thematic analysis [44].

### Dissemination of findings

We will publish the results in the Public Health Research journal in the National Institute for Health Research journal library. We will also submit the study results for publication in an appropriate peer-reviewed journal. We will directly inform our contacts in the Metropolitan Police Service of the findings, and we will also write to trial participants to inform them of the results.

## Discussion

This trial is the first fully powered study, building on our pilot HAVoC trial, to work across agencies for the screening and treatment of depression and/or anxiety in older victims of crime. Older people are particularly vulnerable to the impact of crime because of concurrent life events; family bereavements, physical ill-health, disability, financial difficulties [2, 3, 45]. As the proportion of older people in the UK population rises, society is going to be increasingly impacted by this. Older victims of crime experience significant levels of depression and anxiety, which in many persists for months after victimisation [8]. The VIP trial should provide robust evidence about the effectiveness and cost-effectiveness of treating depression and/or anxiety in this population.

### Research implications

There are virtually no studies published about the mental health of older victims of crime. This trial should demonstrate the utility of a model of cross-agency working in which the police screen older victims of crime, the university team follow-up the victims, and the mental health charity Mind deliver a psychological treatment.

Older victims are particularly hard to reach. Despite significant symptoms of anxiety and depression, which develop rapidly after a crime, existing health care and voluntary services appear unaware of these. If feasible, our model for screening victims, identifying distress and referring for treatment, could be adapted to other research projects in other hard-to-reach and vulnerable victim groups, such as people with intellectual difficulties, in whom mental distress may otherwise be missed.

### Clinical and policy implications

The primary objective of the VIP trial is to determine whether manualised, context-specific CBT, delivered through the mental health charity Mind, is more clinically and cost-effective than TAU for treating anxiety and depression in older victims of crime. Secondary objectives should determine whether older people take up the offer of signposting, and whether health professionals act on this as part of the first step in public health prevention to reduce the potential chronicity of symptoms. The built-in cost-effectiveness analysis should also help evaluate the indirect costs associated with the impact of crime and whether the intervention appears to be cost-effective, such as, for example, reducing the use of health care or social services in this vulnerable population. The final objective is to evaluate whether the model of cross-agency working is successful so that it can then be rolled out across the UK. In this model, the police identify older victims of crime, screen them for distress and, using a coordinator embedded in the police, refer distressed older victims for further evaluation to see whether a CBT intervention is indicated. Delivery of CBT could be undertaken by a variety of agencies, including Mind, IAPT or referral to accredited CBT therapists practising within a GP or community setting.

This trial should provide information on: the proportion of crimes against older people compared to their younger counterparts; the mental health impact that crimes have on older people; the relationship between different types of crime and mental health; the chronicity of anxiety and depressive symptoms associated with a crime; and the health care costs associated with crime. It should also generate evidence to inform guidelines for the Ministry of Justice on how the needs of older victims may be managed.

If our findings suggest that signposting to existing services by the police does not work as part of public health prevention strategy, older victims could be referred directly to mental health services for active surveillance of symptoms, so that an intervention can be delivered if necessary. This framework could still be used nationally and internationally to manage distressed older victims of crime.

## Trial status

This study began recruiting on 1 September 2017 and is currently recruiting, with recruitment estimated to end on 30 April 2021. The current trial protocol is version 8, 17 February 2018.

## Data Availability

The data generated from this study, as well as the consent form and related documents given to participants, will be available from the corresponding author on request.

## Abbreviations

BABCP: British Association of Behavioural and Cognitive Psychotherapies
BAI: Beck Anxiety Inventory
BDI-II: Beck Depression Inventory-II
CACE: Compliers’ average causal effects analysis
CBT: Cognitive behavioural therapy
CTQ-SF: Childhood Trauma Questionnaire Short Form
GAD-2: Generalised Anxiety Disorder 2-item
GAD-7: Generalised Anxiety Disorder 7-item
GP: General practitioner
HAVoC: Helping Aged Victims of Crime
IAPT: Improving Access to Psychological Therapies
ICC: Intra-class correlation coefficient
LSOA: Lower Layer Super Output Area
MINI: Mini-International Neuropsychiatric Interview
MPS: Metropolitan Police Service
NICE: National Institute of Health and Care Excellence
PHQ-2: Patient Health Questionnaire 2-item
PHQ-9: Patient Health Questionnaire 9-item
SNT: Safer Neighbourhood Team
TAU: Treatment as usual
TCC: Therapy Components Checklist
UCL: University College London
VIP: Victim Improvement Package
VIP:TAU: Victim Improvement Package: treatment as usual
